# Noise-Resistant CECG Using Novel Capacitive Electrodes

**DOI:** 10.3390/s20092577

**Published:** 2020-05-01

**Authors:** Chi-Chun Chen, Cheng-Wei Chen, Chang-Wei Hsieh

**Affiliations:** 1 Department of Electronic Engineering, National Chin-Yi University of Technology, Taichung 41170, Taiwan; chichun@ncut.edu.tw (C.-C.C.); s4a713008@student.ncut.edu.tw (C.-W.C.); 2 Department of Photonics and Communication Engineering, Asia University, Taichung 41354, Taiwan

**Keywords:** capacitive electrocardiogram, ambient interference, capacitive electrode, capacitive right leg

## Abstract

For years, capacitive electrocardiogram (CECG) has been known to be susceptible to ambient interference. In light of this, a novel capacitive electrode was developed as an effective way to reduce the interference effect. This was done by simply introducing the capacitive elector in series with a 1 pF capacitor, and the 60 Hz common mode noise induced by AC power lines was cancelled using a capacitive right leg (CRL) circuit. The proposed electrode did as expected outperform two counterparts in terms of SNR, and particularly gave an up to 99.8% correlation between RRIs extracted from an ECG and a CECG signal, a figure far beyond 52% and 63% using the two counterparts. This capacitive electrode was originally designed for long-term noncontact monitoring of heart rate, and hopefully can be integrated to portable devices for other medical care services in the near future.

## 1. Introduction

Electrocardiograms (ECG) were developed as a reliable tool to diagnose heart arrhythmia [1,2]. Additionally, they have been widely employed for monitoring purposes, e.g., in sports training monitoring, fatigue monitoring, mental stress monitoring, and more [3,4,5,6]. A standard lead I ECG requires Ag-AgCl electrode prep pads attached to the skin of a subject via a layer of wet conductive gel. As pointed out in [7,8], conductive gel is in fact a stimulant material, and may even cause allergic reactions. In other words, use of conductive gel is definitely a major disadvantage for long-term ECG monitoring. In light of this, non-contact electrodes have been used over recent years to address the disadvantage of using electrodes in ECG [9]. In contrast, in capacitive ECG (CECG) the signals are capacitively coupled to non-contact electrodes through the clothing worn by a subject as a dielectric layer [10]. A major advantage of CEGG over ECG is that the wet conductive gel required in ECG dries as time elapses, resulting in unwanted noise due to poor contact between electrodes and the skin of a subject. Accordingly, CECG can be well applied to long-term ECG monitoring of bedridden patients. Moreover, CECG can be utilized to diagnose myocardial infarction [11], and can be applied to vital signal monitoring, by means of which sleep-deprived drivers can be alerted [12,13].

As well known, raw CECG signals can be easily overridden or even damaged by a wide variety of noise sources, e.g., power lines [14,15,16], motion artifact [17,18], ambience changes, etc. Though ambience change is actually a common noise source in reality, there have been a limited number of publications on this issue. Hence, the development of a noise-resistant CECG measurement apparatus for diagnosis, and particularly long-term monitoring purposes is a subject of great interest.

Noise accompanying ECG results from intrinsic and extrinsic agents. In the former case, it is mainly the unwanted signals from other factors, or due to motion artifacts, of a subject. Interference from other parts can be filtered out as described in [9,19], while motion artifacts can be removed using an adaptive filter [9,20,21], soft electrodes [17], textile electrodes [22] or a sensor array [23]. In the latter case, it is mainly the common-mode noise, which can be further classified into stationary, e.g., power line noise, and nonstationary types. As presented by Haberman et al. in [14], power line noise, embedded in a CECG, was eliminated using a noise-cancelling circuit. Driven Right Leg (DRL) [24] and Capacitive Right Leg (CRL) [14] were presented as alternative ways to remove the common-mode noise. As compared with a capacitive driven right leg (C-DRL) counterpart, common-mode rejection ratio (CMRR) was significantly raised from 14.4 to 90.22 dB for an improved ECG signal quality, when there was an impedance mismatch between electrodes [25]. They designed this new method to replace the traditional C-DRL model, but our novel contribution is design new active electrodes to enhance raw signals, in order to increase the SNR. The noise effect that all the above-referred work dealt with belongs to the stationary type, while the nonstationary noise effect, due to ambience changes, is a critical issue in CECG measurement, but remains largely unaddressed in the literature. In other words, this issue is addressed herein for the first time. In reality, there exists an equivalent capacitor between a subject and another individual as a noise source [16,26], meaning that the signal carried by the other individual can be capacitively coupled to the subject as either impulsive or stationary noise. Here is a good example of the nonstationary noise interference. When standing or walking alongside a bedridden patient, a caregiver inevitably becomes a moving noise source to an ongoing CECG measurement performed on the patient, and a way must be found to deal with this issue accordingly. This highlights the importance of this work.

In other words, ambient interference must be taken into account when capturing CECG signals. This work aims to develop a noise-resistant solution against the interference due to ambience change, and is presented as two parts. As will be seen later, the noise-resistant performance of the presented capacitive electrode was validated firstly using simulated noise source, and then using human noise sources.

## 2. Materials and Methods

To begin with, the noise effect due to ambience change is modeled in this work. Subsequently, a noise-resistant CECG measurement apparatus is designed using a pair of novel capacitive electrodes and a noise filtering technique.

### 2.1. Common Mode Noise

**Figure 1:**
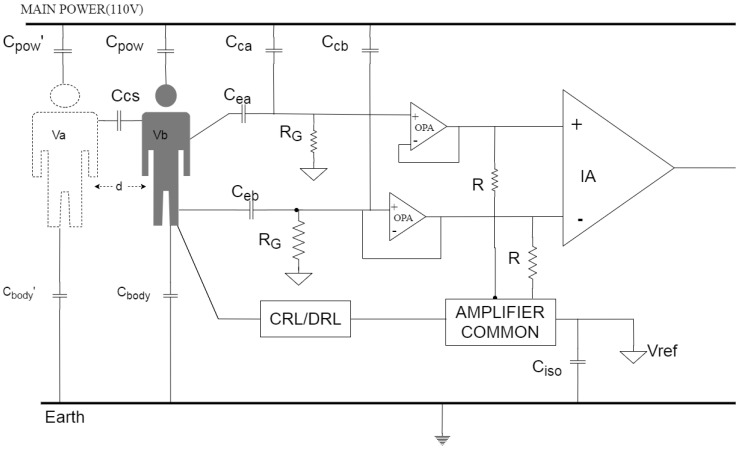
Capacitive coupling between two individuals for an ECG measurement in progress.

As illustrated in Figure 1, there is a capacitive coupling between *individual b*, as a subject for ECG measurement, and *individual a* as a noise source. If the distance d between both is less than the body width, then, the coupling capacitance is given by:

(1) $$C_{\mathrm{CS}}=\varepsilon \frac{A}{d}$$

where ε and A denotes the permittivity of air and the overlapped cross-sectional area between *individuals a* and *b*, respectively.

Respective voltage dividers between a 110 V power line and ground produce a floating body potential V_a_ and V_b_ with an amplitude of 1–2 V. Accordingly, the voltage drop between both is represented as V_ab_, a time-varying signal when *individual b* is on the move. The 1–2 V floating body potential mainly arises from extremely low frequency (ELF) fields which are ubiquitous in our environment, but remain hardly understood. The properties of ELF electric and magnetic fields are described in detail in the World Health Organization’s Environmental Health Criteria 238 on Extremely Low Frequency Fields [27].

The electrostatic charge on *individual a* can be capacitively coupled to *individual b* when *individual a* is moving, leading to an interference current on the ECG measurement, expressed as:

(2) $$i_{\mathrm{CS}}=C_{\mathrm{CS}}\frac{\mathrm{dV}}{\mathrm{dt}}$$

As expressed in Equation (2), C_cs_ is inversely proportional to *d*, and Equation (2) reveals that i_cs_ varies directly as C_cs_. It means that an ECG measurement is strongly interfered with by *individual b* staying close to the subject.

Besides, it is presumed that the approaching velocity of *individual a* toward *b* has an effect on the ECG measurement. This is simply for the reason that i_cs_ varies directly as dv/dt and c_s_. Since dv/dt increases with the approaching velocity, a large current is coupled from *individual a* to *b* accordingly.

### 2.2. Electrode Design

**Figure 2:**
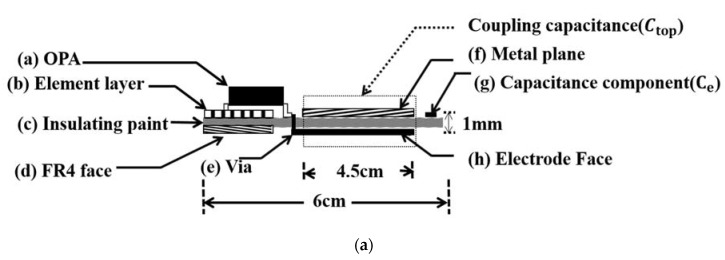

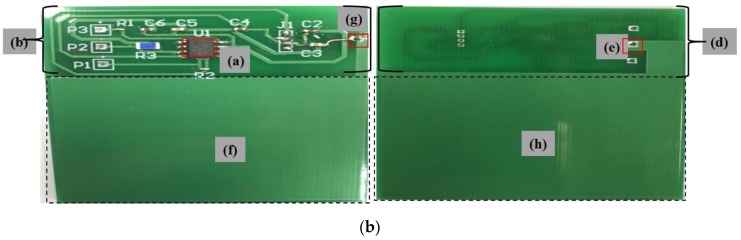
(**a**) Cross-section and (**b**) photos of the presented capacitive electrodes.

As illustrated in Figure 2, the novel capacitive electrode comprises a stack consisting of (a) an operational amplifier (OPA), (b) an element layer, (c) a dielectric layer, (d) an FR4 face layer on the left side, (e) a via hole in the center, and (f) another stack composed of a metal layer, (g) an external capacitor C_e_, (h) an contact layer on the right side.

The 1 mm thick dielectric layer is sandwiched by the metal and the contact layers, both measuring 4.5 × 4.5 cm^2^, and a parallel plate capacitor C_top_ is formed in a dotted line frame accordingly. The dielectric layer has a dielectric constant of 4.4, that is, ε = ε_r_ε_0_ = 4.4 × 8.85 × 10^−12^, and then C_top_ is evaluated as 78.95 pF using:

(3) $$C_{\mathrm{top}}=\varepsilon_{0}\varepsilon_{r}\frac{A}{d}$$

#### 2.2.1. Electrode Equivalent Circuit

**Figure 3:**
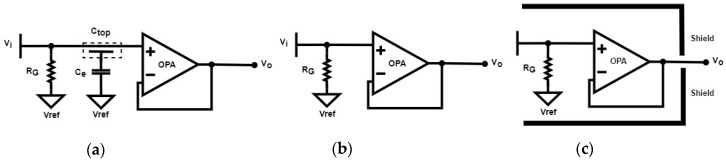
Three CECG electrodes. (**a**) The proposed electrode, (**b**) a high input impedance electrode and (**c**) the one in (**b**) is enclosed with a guard ring for noise shielding.

As illustrated in Figure 3a, C_top_ in Figure 2a is connected to V_ref_ via a 1pF capacitor C_e_. The equivalent impedance seen by the source V_i_ is expressed as:

(4) $$Z=\frac{1}{2\cdot \pi \cdot f\cdot \left(C_{e}//C_{\mathrm{top}}\right)}//R_{G}$$

The RC low pass filter accepts the low-frequency physiological signals of interest, and rejects unwanted high-frequency common mode noise. In this manner, the capacitively coupled current, i.e., the nonstationary noise, from *individual a* partially bypasses R_G_ to V_ref_ via C_e_, and then the interference can be reduced significantly. Two CECG electrodes, as illustrated in Figure 3b,c were existing solutions reported in the literature which were rebuilt as counterparts. The one in Figure 3b has a high input impedance, and is most commonly employed in CECG measurements [9]. The one in Figure 3c is enclosed by a guard ring to reduce noise interference [10]. For convenience of comparison, the electrodes in Figure 3a–c are hereafter referred to as electrodes 1–3, respectively.

#### 2.2.2. CRL Circuit

**Figure 4:**
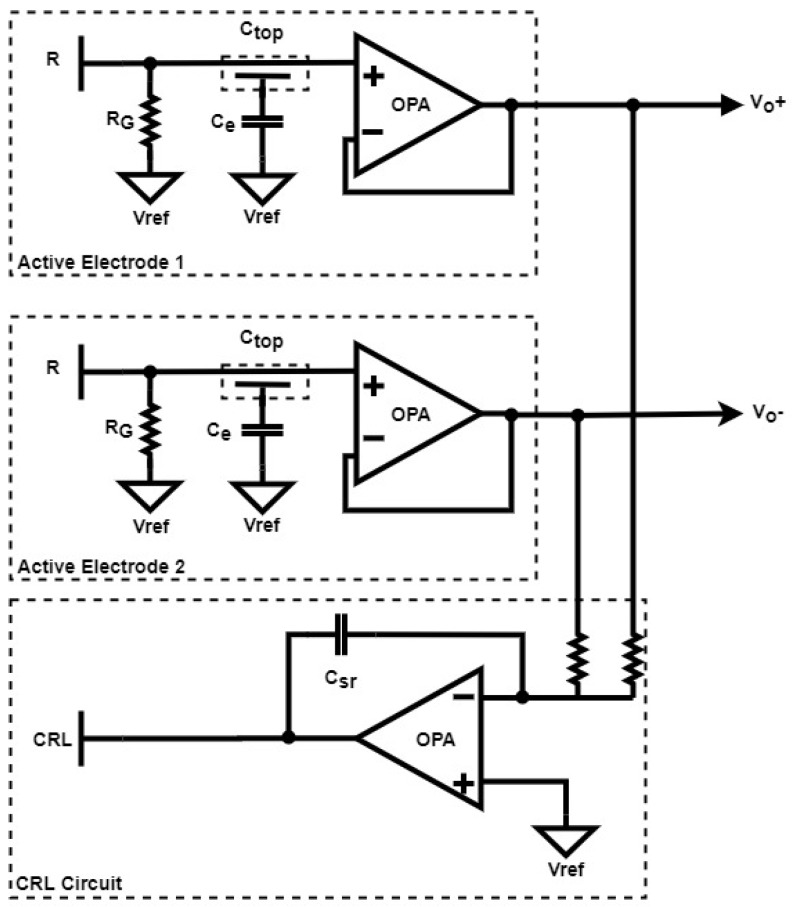
Common-mode noise cancelling circuit.

A capacitive right leg (CRL) circuit, as illustrated in Figure 4, aims to reject the 60 Hz common mode noise due to AC power line contamination. This is done using the inverted common mode signals which are fed into the body of a subject via a capacitive electrode. As illustrated in Figure 4, a resistor of R = 10 K and a capacitor of C_sr_ = 1 pF are employed not only to block the low-frequency physiological signals, but also to amplify the common mode noise for signal cancellation, leading to an improvement in ECG quality.

### 2.3. Signal Acquisition

**Figure 5:**
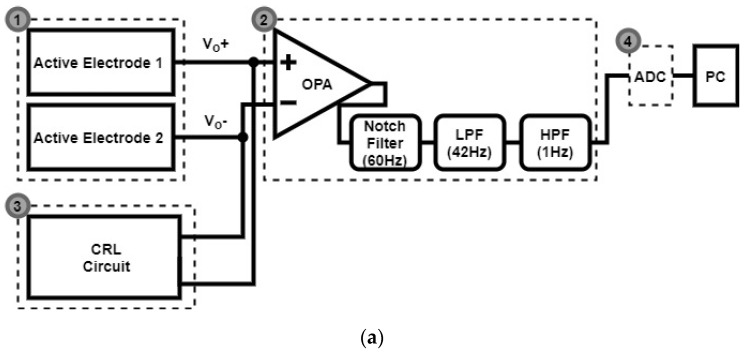

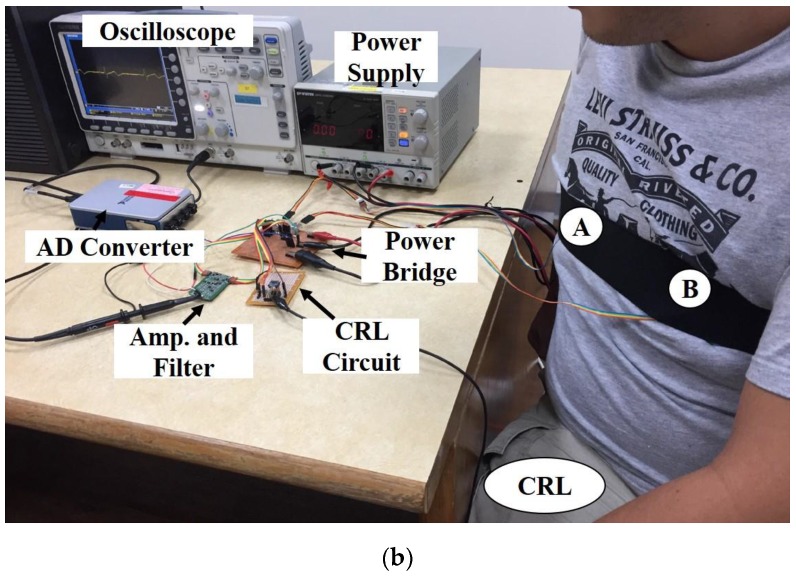
(**a**) Block diagram and (**b**) a photo of the presented CECG measurement apparatus.

As illustrated in Figure 5a, the presented non-contact ECG measurement apparatus mainly involves four parts, namely: (1) a pair of active electrodes, (2) a cascade of an instrument amplifier, a 60 Hz notch filter, a low pass filter and a high pass filter, (3) a capacitive driven right leg circuit and (4) an AD converter. The specifications of the ECG in general are that the LPF cutoff frequency is 50 Hz and the HPF cutoff frequency is less than 10 Hz (the specifications are shown in [16]). Accordingly, the LPF was designed to have a corner frequency of 42 Hz, and the HPF was to have 1 Hz. The output pins, labelled V_o_^+^ and V_o_^-^, in Figure 4 are connected to the AD623 instrumentation amplifier in Figure 5a. As presented in Figure 5b, the pair of capacitive electrodes in part 1 are attached to the chest of a subject on spots A and B, and the CRL electrode is attached somewhere on the right leg, forming a Lead I ECG. The sensed ECG signals are instantly taken by part 2, and then transmitted to a PC for subsequent signal processing using MATLAB.

### 2.4. Performance Validation

A testbed was built to assess the noise-resistant performance of this work, and a performance comparison was then made among this proposal and two counterparts, as illustrated in Figure 3a–c.

#### 2.4.1. Noise-resistant Performance Testbed

The testbed is made up of four parts, namely: (1) a simulated ECG signal source, (2) a signal acquisition and noise filtering block, (3) an AD converter and (4) a simulated noise source.

##### Simulated ECG Signal Source

**Figure 6:**
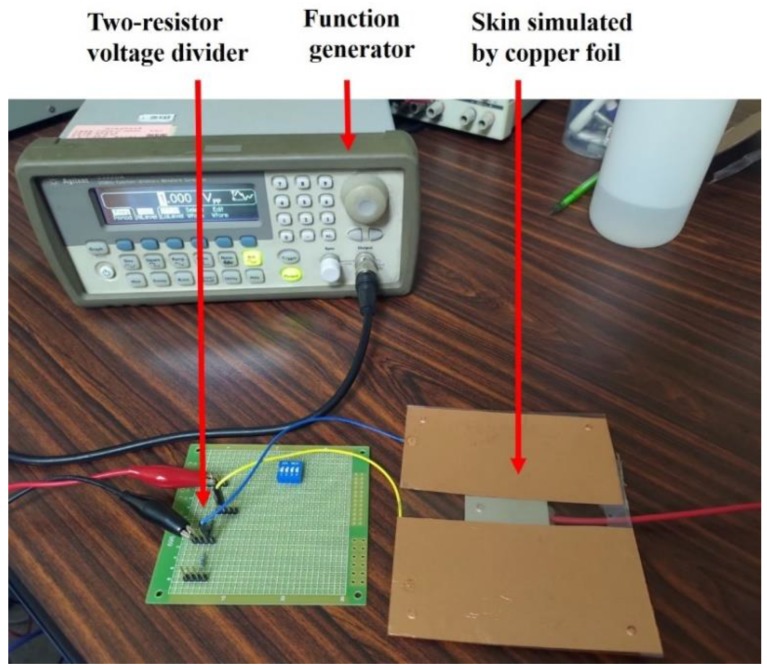
A simulated ECG signal source using an Agilent 33220A function/arbitrary waveform generator and a two-resistor voltage divider.

Presented in Figure 6 is a photo of the simulated ECG signal source, in compliance with IEC 60601-2-47 [28].

The collective heart-electrode-ground route is modeled as a two-resistor voltage divider, consisting of a 100 KΩ and a 100 Ω resistor, and heart waveform is simulated as a 1Vp-p ECG signal generated by an Agilent 33220A function/arbitrary waveform generator. Accordingly, a signal attenuation of 20 × log(1001) = 60 dB is provided by the voltage divider.

**Figure 7:**
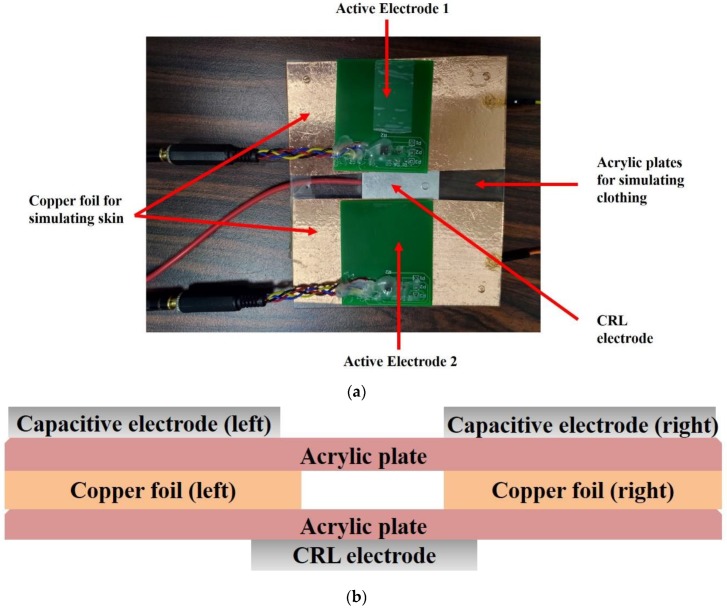
(**a**) A top view photo of the proposed electrode, and (**b**) cross sectional view of (**a**) where two pieces of aluminum foil, sandwiched between the acrylic plates, are employed to simulate the skin of a subject.

Presented in Figure 7 is a photo of a pair of capacitive electrodes together with the CRL electrode and the built measurement circuit. The clothing worn by a subject is simulated as two acrylic plates, each measuring 5 × 5 cm^2^, whereon two capacitive and a CRL electrode are supported. The skin is also simulated as two pieces of copper foil sandwiched between the acrylic plates. The attenuated signal is then applied across the two pieces of copper foil for CECG measurement. Here, it must be pointed out that there must be a gap between the two pieces of copper foil, say 2 cm in this case, or the signal source will be short circuited.

##### Signal Capturing and Noise Filtering

**Figure 8:**
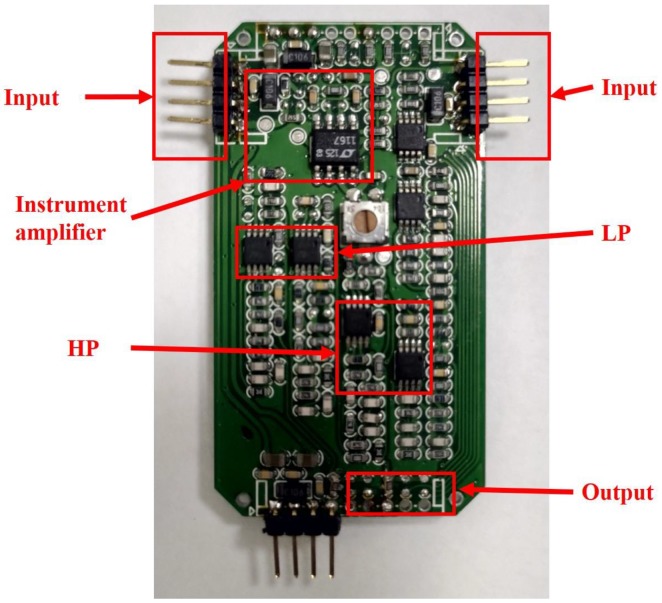
Block configuration and I/O terminals.

Presented below (Figure 8) is a photo of the circuit implemented on a PCB. As explicitly illustrated in Figure 5a, a pair of active electrodes are followed by an instrument amplifier for ECG signal amplification, a 60 Hz notch filer, a LPF and a HPF for signal filtration.

##### AD Converter

Analogue to digital signal conversion is performed using an NI cDAQ-9171 USB chassis. It is equipped with hot plug modules, and has four 32-bit general-purpose counters/timers. As explicitly illustrated in Figure 5a, the HPF is followed by an ADC, meaning that the output terminals of the circuit on the PCB shown above are connected to one of the four channels in the NI cDAQ-9171 USB chassis in reality, and data are acquired using NI-DAQmx and LabVIEW. Subsequently, an analysis on the received ECG signals is made using MATLAB.

##### Simulated Noise Source

**Figure 9:**
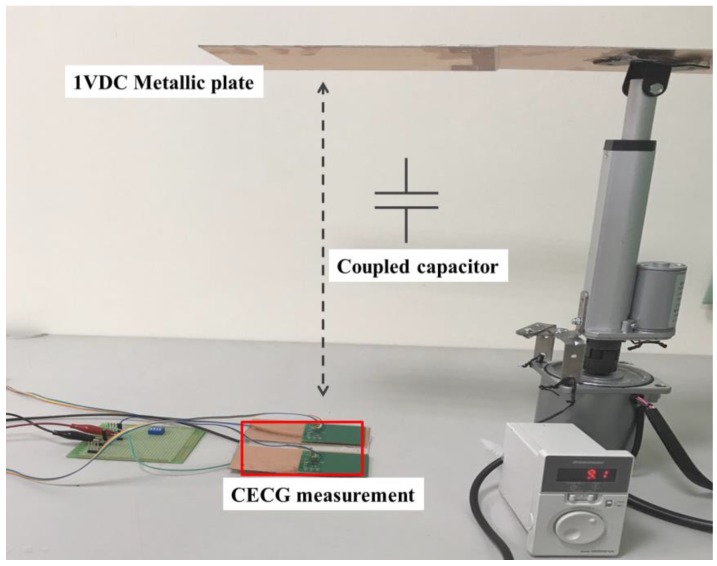
A photo of the built noise-resistant performance testbed.

Presented in Figure 9 is a photo of the built testbed. As illustrated therein, a motor-driven metallic plate is placed at the top, and the voltage thereon is kept constant at 1 VDC as a noise source. The plate can be instructed to move either downward or upward to investigate the noise effect on ECG measurements. The unit, shown in Figure 8, is framed in red, placed on the work table, and is oriented parallel with the overhead metallic plate, as indicated by a double arrow line in between. Once the plate moves, the charge on the plate is capacitively coupled to the electrodes. In this context, the effect of noise source, either on the move or at rest, can be deeply investigated using this testbed.

#### 2.4.2. Experimental Protocol

(1) Simulated noise cases

Experiments were conducted in two cases. In the first case, the noise source, i.e., the motor-driven metallic plate in Figure 9, was initially and respectively placed 90, 60 and 30 cm above the work table. It was then instructed to move downward at 30 cm/s for 1 s, that is, a displacement of 30 cm, and move upward back to the initial height, say 90 cm, at the same speed. This 2-s cycle was repeated four times, that is, for a duration of 10 s. Then, the plate moved downward to the next interval, say [30, 60 cm]. In the second case, the noise source instead lay at a height of 90 cm above the table, and was then commanded to move downward at 0.1, 0.4 and 0.7 m/s respectively. At each speed, the experiment was conducted three times. Noise resistant performance was compared among the counterparts. Here, it must be stressed that non-noisy ECG signals were generated by the Agilent 33220A function/arbitrary waveform generator presented in Figure 6.

Next, the metallic plate was instructed to approach the subject at a constant speed, and signal-to-noise ratio (SNR) [20] is defined for quantitative comparison as:

(5) $$SNR=20\log\frac{P\left(R_{peak\;}\right)}{P\left(\mathrm{noise}\right)}$$

where P(*R_peak_*) is the power of the 100-ms ECGs centered on the detected R peak and P(noise) is the power of the ECGs outside those 100-ms ECGs centered.

(2) Human noise cases

Experiments were conducted in a slightly different way than in the previous case, that is, the metallic motor-driven plate was replaced with an individual this time, who moved toward another one, but seated on a chair, as a subject for CECG measurements. Besides, the noise source approached the subject in somewhat different ways as well. As presented in Figure 5b, the electrodes were pressed tightly against the chest of the subject. Experimental protocol is detailed as follows, and, as before, noise resistant performance was compared in two cases among the counterparts.

**Figure 10:**
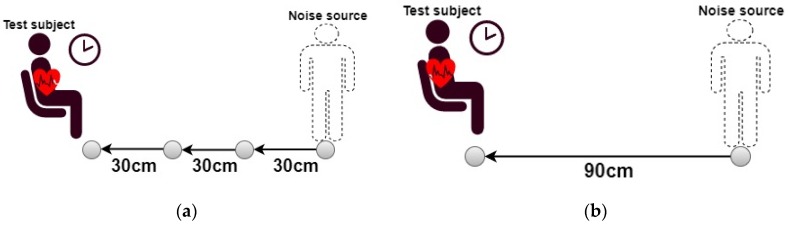
The noise source (**a**) takes 30 cm steps, and (**b**) approaches at constant speeds toward the subject.

As illustrated in Figure 10a, the noise source initially stood 90 cm away from the subject and remained still until the measured CECG signal became well stabilized. Then, the noise source took a 30 cm step toward the subject, during which the noise interference was observed, and both were now 60 cm apart. The noise source took a step back to the start point. The back and forth cycle was repeated and endured 10 s. Next, the noise source moved forward, and stood 60 cm away from the subject. As before, the back and forth cycle was repeated but in the interval [30, 60 cm] this time. The experiment was repeated until the noise source reached the subject. As expected, a nearby noise source caused stronger interference to CECG signals than a remote one.

As illustrated in Figure 10b, both individuals were initially 90 cm apart. It is expected that the capacitively coupled current increases with the velocity of the moving noise source. For performance testing of the presented electrodes, the noise source was made to approach the subject at a low speed between 0.1 and 0.3 m/s, a middle speed between 0.31 and 0.6 m/s and a high speed between 0.61 and 0.9 m/s. At each speed, this noise interference experiment was conducted three times.

For discussion purposes, waveform I hereafter refers to a CECG signal captured in phase 1 using electrode 1, and so on. For accurate comparison, experiments in each phase were carried out under the same conditions as much as possible. In other words, electrodes were placed on the same spots of a subject, in either the simulated or the human noise cases, and noise sources interfered with the experiments in the same manner.

Finally, the performance of the electrodes are compared in terms of RRI between an ECG and three captured CECG signals. Signals were coupled to the electrodes on two chest spots via a layer of clothing, and captured *under the same conditions as much as possible*. Meanwhile, the noise source interfered with the measurement in a similar way as before, that is, the noise source remained still over the intervals [0, 10 s] and [70, 80 s], but kept walking over the interval [10, 70 s].

## 3. Results

### 3.1. Simulated Noise Cases

#### 3.1.1. Noise Effect Versus Distance

**Figure 11:**
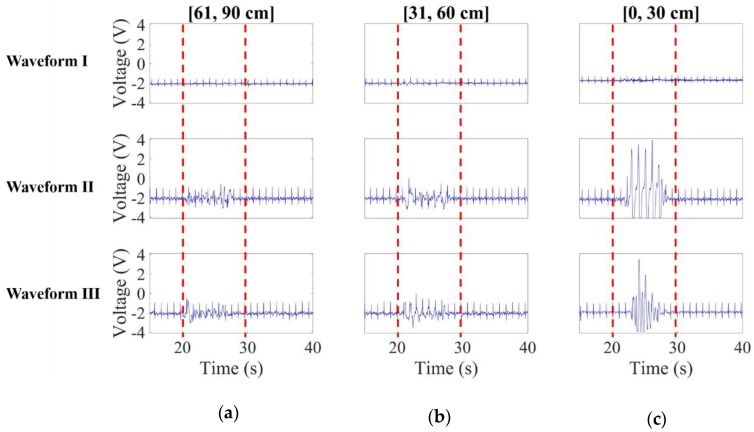
CECG waveform comparison when the simulated noise source approaches the subject at 30 cm/s over intervals (**a**) [61, 90 cm], (**b**) [31, 60 cm] and (**c**) [0, 30 cm].

The red framed parts in Figure 11a–c are made for comparison among *waveforms* I–III for the metallic motor-driven plate approaching the subject at 30 cm/s over the intervals [61, 90 cm], [31, 60 cm] and [0, 30 cm], respectively. As requested, the CECG signals in all cases before and after the red frames stayed well stabilized. As can be found in Figure 11a, *waveform* I obviously outperforms the counterparts in terms of noise resistant performance.

Subsequently, the metallic motor-driven plate further approached the subject. As before, the red framed CECG signals in Figure 11b,c were for the [31, 60 cm] and [0, 30 cm] cases, respectively. As expected, a significantly higher level of noise interference was observed in waveform III in Figure 11b than Figure 11c, and, as intended, the presented electrodes well outperforms the counterparts again in both cases in respect of the noise resistant performance

**Figure 12:**
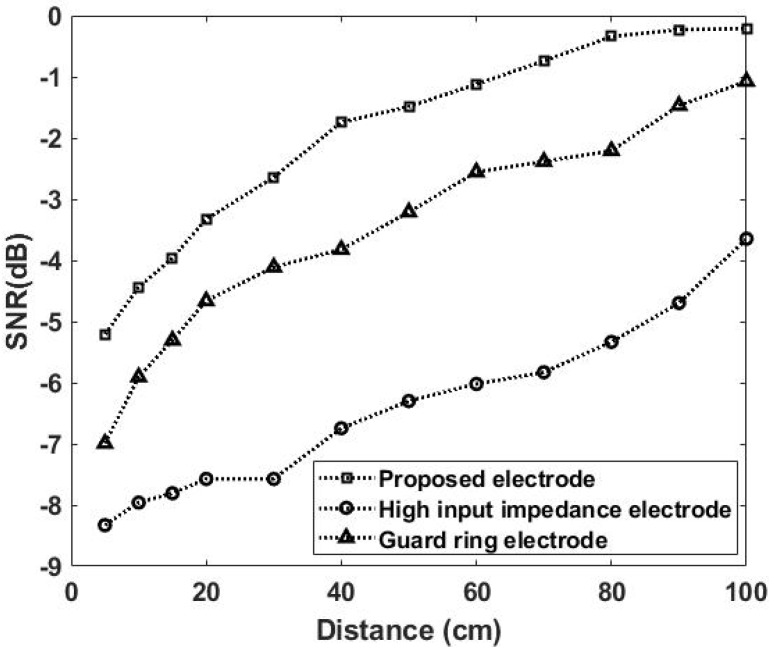
Comparison of SNR versus the distance between the subject and the simulated noise source.

Figure 12 gives a comparison of SNR versus the distance in between the noise source and the subject. As expected, SNR increases with the distance, and, more importantly, this work is experimentally validated to rank the first in terms of SNR.

#### 3.1.2. Noise Effect Versus Speed

**Figure 13:**
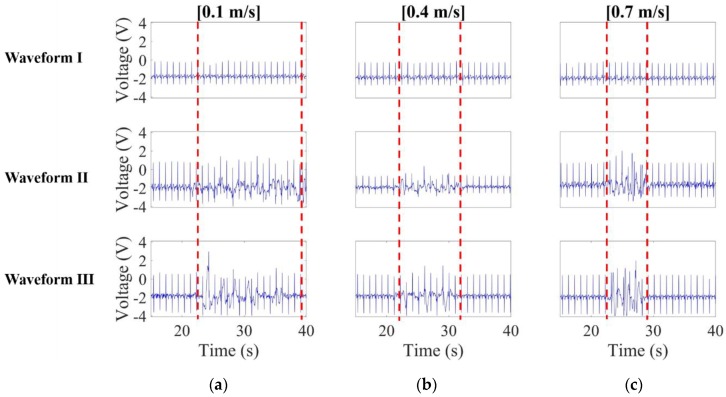
CECG waveform comparison when the simulated noise source approaches the subject at (**a**) 0.1 m/s, (**b**) 0.4 m/s and (**c**) 0.7 m/s.

The effect of noise source velocity is investigated here. Presented in Figure 13a–c are the comparisons on the noisy CECG signals for the noise source approaching at 0.1 m/s (low speed), 0.4 m/s (middle speed) and 0.7 m/s (high speed), respectively.

It is noted that *waveform* I, unlike *waveforms* II and III, remains virtually unaffected by noise, even though there are furious fluctuations in *waveform* II, and even signal saturation in *waveform* III in Figure 13b. Hence, the presented bypass capacitor design is experimentally validated as effective against capacitively coupled noise from a moving source.

**Figure 14:**
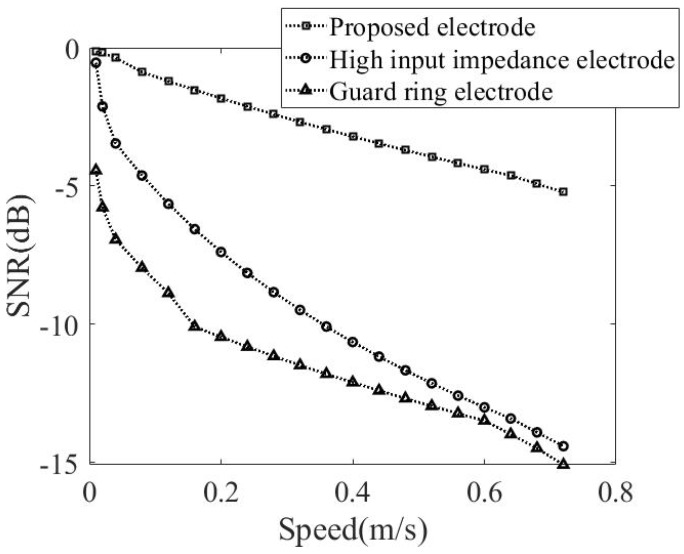
Comparison of SNR versus the speed at which the simulated noise source approaches the subject.

In the first place, a noise-free benchmark is established in exactly the same way as in the previous case. Next, the simulated noise source was commanded to move at different speeds for the SNR computation. Figure 14 gives a comparison of SNR versus the noise source speed. It is noted here that SNR decreases with the speed, in agreement with the fact that the level of noise interference increases with the speed. Besides, the presented bypass capacitor design is found to outperform the counterparts again, particularly at high speeds.

### 3.2. Human Noise Cases

#### 3.2.1. Noise Effect Versus Distance

**Figure 15:**
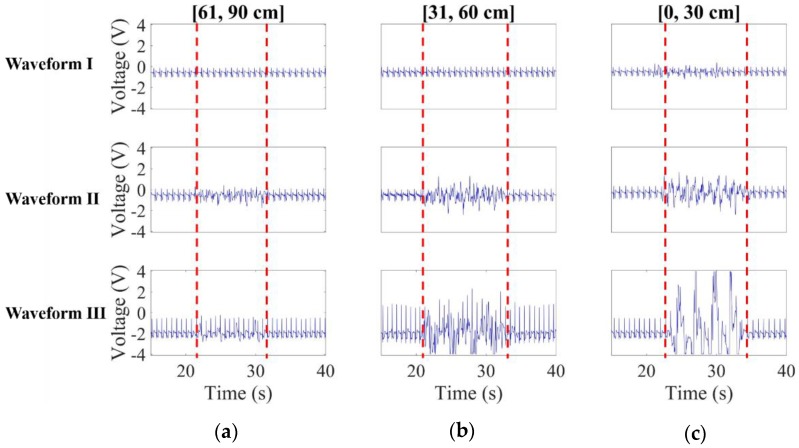
CECG waveform comparison when the human noise source approached the subject at 30 cm/s over intervals (**a**) [61, 90 cm], (**b**) [31, 60 cm] and (**c**) [0, 30 cm].

Red framed in Figure 15a are the measured noisy CECG signals. 

Obviously, waveform I remains less affected by noise than waveforms II and III. As can be found in Figure 15b,c, waveform I apparently remains the best again in terms of noise suppression, and waveforms II and III in Figure 15a outperform the counterparts in Figure 15b,c in terms of signal fluctuation and saturation. This makes sense, since a nearby noise source is expected to cause a higher level of interference than a remote one.

#### 3.2.2. Noise Effect Versus Speed

**Figure 16:**
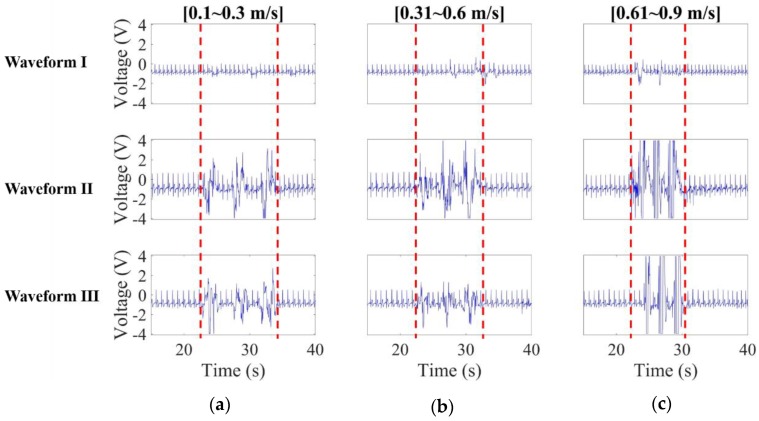
CECG waveform comparison when the human noise source approached the subject at (**a**) 0.1–0.3 m/s (low speed), (**b**) 0.31–0.6 m/s (middle speed) and (**c**) 0.61–0.9 m/s (high speed).

In this case, measured noisy CECG signals were presented in Figure 16. As expected, higher/lower frequency noise is obviously seen in waveforms II and III in the high/low speed cases. Waveform I remains virtually unaffected by noise in the low speed case, while slightly affected in the middle and high speed cases. In short, noise interference can be suppressed to a great extent using the presented electrodes.

**Figure 17:**
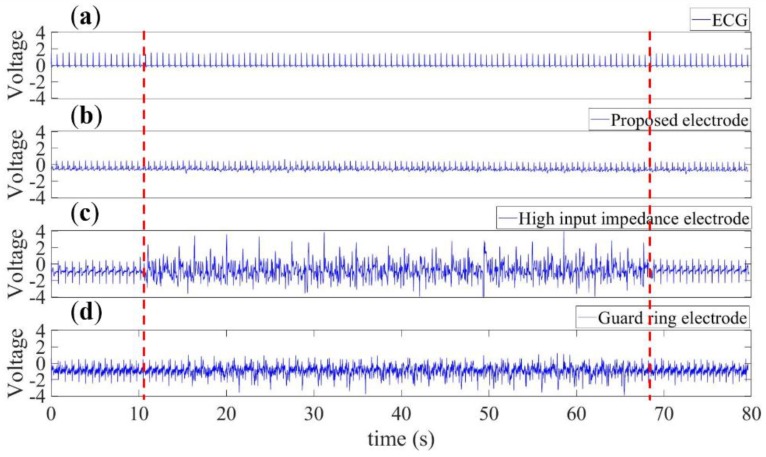
The ECG signal in (**a**) was measured as a benchmark, and the CECG signals in (**b**–**d**) were for comparison purposes using the electrodes in Figure 3a–c, respectively. There is a 99.8% correlation between RRIs extracted from the red framed signals in (**a**) and (**b**), 52% between (**a**) and (**c**), and 63% between (**a**) and (**d**).

As before, red framed in Figure 17a–d are the measured noisy ECG and CECG signals using the electrodes in Figure 3a,b and Figure 7, respectively.

Here, it must be pointed out that the repetitive ECG signal in Figure 17a was captured all the way through phases 1–3. It is clearly seen that the red framed signal in Figure 17b was far more noise-resistant than those in (c) and (d). Quantitatively speaking, there is a 99.8% correlation between the RRIs extracted from the waveforms in the red framed intervals in Figure 17a,b, 52% between Figure 17a,c, and 63% between Figure 17a,d. This highlights a feature of this work over its counterparts.

## 4. Discussion

CECG has long been known to be susceptible to noise. In light of this, this paper presents a novel capacitive electrode as a solution to high noise susceptibility, and SNR can be improved accordingly. Long-term CECG monitoring can be made for home care of bedridden patients, and early stage cardiovascular disease can be diagnosed using this technique as well.

Taking advantage of this technique, there is a high correlation between the RRIs provided by CECG and standard ECG, meaning that CECG can serve as an alternative to standard ECG. The presented capacitive electrode was experimentally validated herein to outperform counterparts in terms of noise resistant performance. As explicitly stated previously, this work has been experimentally validated to outperform two conventional capacitive counterparts, and more importantly there is a correlation up to 99.8% between RRIs provided by CECG and standard ECG. In other words, it can serve as an alternative to standard ECG, particularly for long-term ECG monitoring.

Because of the susceptibility of CECG to noise tests must be conducted under well-conditioned conditions, i.e., a single noise source was taken into account at a time for comparison purposes. This paper presents novel capacitive electrodes, and the noise-resistant performance of CECG measurement apparatuses was tested under well-controlled conditions, that is, a single moving noise source was taken into account at a time for comparison purposes. The noise source was firstly simulated as a metallic motor-driven plate, and then as a moving individual. CECG signal saturation, due to strong capacitively coupled noise, was found in counterparts, while instead excellent noise-resistant performance was observed in this work. In other words, the presented capacitive electrodes are believed to be an advantageous candidate when applied to CECG devices. A key issue in this work is to build a pair of completely matched (identical) electrodes, such that common mode noise can be rejected as intended.

The limitation in this preliminary study should be addressed in future studies. Due to the methodological problems in CECG studies, we tentatively put forward as a demonstration the one-shot case of the actual human signals, but not a group comparison. As a matter of fact, CECG measurements were performed with multiple experiments on one subject. All the results are consistent and reproducible to an extent. For illustrative purposes, only the waveforms captured from one of the results were presented in Figure 15 and Figure 16. In the future, for the purpose of improving this study, a good experimental design for group comparison should be considered, such as a counterbalanced measurement design.

## 5. Conclusions

This paper presents novel capacitive electrodes, based on which a CECG measurement apparatus was built accordingly. Noise effect was deeply investigated, and other issues, including noise filtering, signal processing and more, were all addressed herein as well. Besides, a testbed was also built to test the noise-resistant performance of a CECG measurement apparatus. As it turns out, this work has been well validated to outperform counterparts using the testbed, and may be viewed as a benchmark for the further development of CECG measurement apparatuses.
